# Microfabricated Tactile Sensors for Biomedical Applications: A Review

**DOI:** 10.3390/bios4040422

**Published:** 2014-11-03

**Authors:** Paola Saccomandi, Emiliano Schena, Calogero Maria Oddo, Loredana Zollo, Sergio Silvestri, Eugenio Guglielmelli

**Affiliations:** 1 Center for Integrated Research, Unit of Measurements and Biomedical Instrumentation, Università Campus Bio-Medico di Roma, Via Álvaro del Portillo, Rome 21-00128, Italy; E-Mails: p.saccomandi@unicampus.it (P.S.); s.silvestri@unicampus.it (S.S.); 2 The BioRobotics Institute, Scuola Superiore Sant’Anna, Polo Sant’Anna Valdera, Viale Rinaldo Piaggio 34, Pontedera (PI) 56025, Italy; E-Mail: oddoc@sssup.it; 3 Center for Integrated Research, Laboratory of Biomedical Robotics and Biomicrosystems, Università Campus Bio-Medico di Roma, Via Álvaro del Portillo, Rome 21-00128, Italy; E-Mails: l.zollo@unicampus.it (L.Z.); e.guglielmelli@unicampus.it (E.G.)

**Keywords:** tactile sensors, microfabrication, medicine, prosthetic hands, artificial skin, biomechanical measurements, microsurgery, endoscopy

## Abstract

During the last decades, tactile sensors based on different sensing principles have been developed due to the growing interest in robotics and, mainly, in medical applications. Several technological solutions have been employed to design tactile sensors; in particular, solutions based on microfabrication present several attractive features. Microfabrication technologies allow for developing miniaturized sensors with good performance in terms of metrological properties (e.g., accuracy, sensitivity, low power consumption, and frequency response). Small size and good metrological properties heighten the potential role of tactile sensors in medicine, making them especially attractive to be integrated in smart interfaces and microsurgical tools. This paper provides an overview of microfabricated tactile sensors, focusing on the mean principles of sensing, *i.e*., piezoresistive, piezoelectric and capacitive sensors. These sensors are employed for measuring contact properties, in particular force and pressure, in three main medical fields, *i.e*., prosthetics and artificial skin, minimal access surgery and smart interfaces for biomechanical analysis. The working principles and the metrological properties of the most promising tactile, microfabricated sensors are analyzed, together with their application in medicine. Finally, the new emerging technologies in these fields are briefly described.

## 1. Introduction

The first interest in the touch-sensing technology arose between the end of 1970s and the beginning of 1980s, when some researchers started investigating its role in the field of robotics [1,2].

Harmon published a survey on tactile sensors in 1981, analyzing the requirements that such transducers have to fulfill in many technological areas, such as military, agriculture, manufacturing and medical industries, and the expectation of the market regarding their performance and their potentiality with respect to robotic needs [3,4,5]. He defined tactile sensing as “continuously variable touch sensing over an area where there is special resolution”, and predicted the robotic industry and the prosthetic and orthotic applications to be the main future areas of interest.

In recent decades, robotics is rapidly growing, particularly in the medical field. The features provided by robotic tools in medicine showed their usefulness in many applications, such as in the development of an efficient sense of touch emulating the human sensory system [6,7], or in the design of surgical [8,9,10] and endoscopic [11] tools. These tools are designed to help the clinician perform habitual or difficult procedures, and are often required for compensating some characteristics of human ability and dexterous movements. Therefore robotic devices should guarantee dependability and safety, and be bio-inspired, in order to preserve and enhance most of the natural features which characterize human behavior.

Among these natural features, the sense of touch attracts huge attention. If a task is achieved using a robotic manipulator, sensory inputs similar to those possessed by humans are essential to provide the necessary feedback to explore and interact with objects. Tactile sensors are responsible of contact information for robotic tools and devices.

Simultaneously, the microfabrication technology is gathering more and more interest [12]. The feasibility to fabricate objects with dimensions in the range of micrometer to millimeter promoted the spread of this technology in several fields of science, with particular regards to medicine.

Several valuable features (e.g., small size, high sensitivity, accuracy and precision, low power consumption) coupled with the chance of providing a better outcome for the patients and lower health care cost, strengthen the potential of micro-fabricated devices in medical applications. Hence a wide variety of applications in surgical, diagnostic devices and therapeutic areas, is involved in the continuous expansion of microfabricated devices [13]. The majority of micro-electro-mechanical systems (MEMSs) implemented in biomedical applications are sensors for monitoring physical parameters such as pressure, acceleration, fluid flow, temperature. They are commonly used in orthopedic research field in the study of muscles and patient’s posture, in the monitoring of blood flow and in implanted microsystems [14], in microsurgery [15], bladder and intraocular applications [16] and in measurement of cerebro-spinal fluid pressure [17]. They are also employed in long term monitoring of prosthetic devices, in respiratory monitoring to measure gas flows in spirometric devices and mechanical ventilators [18,19], in microfabricated drug delivery devices [20] and in the detection and characterization of tumor cells from blood [21,22].

Many features required by artificial tactile sensing can be achieved by using microfabricated devices, such as the reduced size and high spatial resolution, the high surface area to volume ratio, the flexibility, the small response time due to the reduced mass, the possibility of integration in soft surfaces, low power consumption and the reduced electronic circuit.

Some exhaustive review papers regarding tactile sensors for medical application have been previously published. The paper of Lee was one of the most complete reviews within the nineties [23], followed by the paper of Eltaib about tactile sensing technology for minimally invasive surgery in 2003 [24] and the paper of Tiwana and colleagues in 2012 [25], concluding with the review of Lucarotti and colleagues about bio-artificial tactile sensing [26]. The authors recommend these reviews, so as to guide the reader to the wider panorama of main technologies and applications for artificial tactile sensing. Our review aims to go beyond the perspectives of the previous works, focusing on the areas in which microfabrication has made an impact on artificial tactile sensors. In particular, we investigated the main microfabrication technologies used to develop tactile sensors for biomedical purposes (prosthetics and artificial skin, minimal access surgery, smart interfaces for biomechanical measurements) including information about multimodal sensors and hints about the new frontiers in this field. Our analysis of the literature is carried out to emphasize the application of microfabrication in this field, exploiting the wide scenario of medical applications of tactile sensors.

The paper is organized as follows: firstly, three widely used technologies for microfabricated tactile sensors have been reported—*i.e*., piezoresistive, piezoelectric and capacitive sensors—and hints for the principles of microfabrication are described. Subsequently, the applications of micromachined tactile sensors are illustrated for each field, along with a brief description of multimodal tactile sensors and of new technologies, with special attention to optical and microfluidical ones.

## 2. Principles of Measurement

### 2.1. Piezoresistive Sensors

Piezoresistors refer to resistors whose resistivity changes with strain, due to an applied force [27], such as semiconductor silicon; their sensing principle differs from the one of strain gauge, whose resistance change with strain because of shape deformation. In piezoresistors, the resistivity *ρ* is defined as:

(1) $$\rho =\frac{1}{n\cdot q\cdot \mu }$$

where n is the number of charge carriers, q is the charge per single charge carrier and *µ* is the mobility of charge carriers, expressed as:

(2) $$\mu =\frac{q\cdot <t>}{m^{*}}$$

where, <*t*> is the mean free time between two carrier collision events, and m* is the effective mass of a carrier in the crystal lattice. Both <*t*> and m* are related to the average atomic spacing in the lattice, which is subject to changes under physical strain and deformation [28]. The resistive element is, usually, an elastomer, a conductive rubber, a conductive ink or a carbon fiber that is sensitive to pressure. In general, piezoresistive sensors are affordable and characterized by good sensitivity; as a matter of fact, the simple working principle allows them to be easily microfabricated and to have a simple electronics, and there is no significant noise due to crosstalk. As an example, usually the Wheatstone bridge configuration is used to convert the change of resistance into corresponding voltage output [29]. The main drawback is related to the hysteresis and low frequency response, compared with capacitive sensors [25].

### 2.2. Piezoelectric Sensors

Piezoelectric sensors are based on the direct piezoelectric effect, *i.e.*, the change of electrical polarization of the element undergoing mechanical deformation [30,31]. Many materials show piezoelectric properties, such as some crystal (e.g., quartz, berlinjite, turmaline) and ceramics (e.g., Lead Zirconate Titanate—PZT), and other materials (e.g., Zinc Oxide and Polyvinylidene Fluoride—PVDF). In a piezoelectric material, the link between mechanical stress and strain and the electric field and electric induction under general conditions is described by a system of equations that is reported and discussed in [31].

Piezoelectric sensors are preferred in case of measurement of vibration, since they are characterized by good high frequency response. Among many piezoelectric materials, the organic ferroelectric ones are preferred. In particular, PVDF is a good material to be used in tactile sensors due to its particular features, such as high piezoelectric voltage sensitivity, flexibility, and lightness, responsiveness over a wide frequency range and inertness to chemical agents [32]. In addition, its copolymer with trifluoroethylene, the P(VDF/TrFE), is deeply investigated for tactile sensing applications, because of its high cristallinity which improves the piezoelectric properties (e.g., d_33_ values −38 pC·N^−1^ for P(VDF-TrFE) *versus* −33 pC·N^−1^ in pure PVDF [33,34]). Recently, the piezoelectric behavior of vinylidene fluoride (VDF) oligomer, a new substance that has a smaller number of VDF oligomers and a lower molecular weight than PVDF, was evaluated to be suitable for tactile purposes, thanks to the potential to be miniaturized [35,36].

The main concern is related to the high internal resistance, entailing piezoelectric sensors to be significantly affected by the input impedance of the readout electronic circuitry, and the sensitivity to temperature influence. Piezoelectric sensors are characterized by excellent dynamic behavior, but low sensitivity to static forces [37]. Indeed, output charge of the PVDF sensor exponentially decrease with time due to the leakage current of the sensor, depending on its internal impedance and on the impedance of the readout electronics. As a relevant industrial example of system dimensioning to measure quasi-static forces, the reader could refer to application notes and charge amplifiers provided by Kistler [38,39].

### 2.3. Capacitive Sensors

Capacitive sensors are made of two conductive plates with area A, placed at distance *d* each another, with a dielectric material between them. Two possible configurations can be adopted to realize capacitive tactile sensors, based on displacement principia: (1) the change of overlapping area A between the two plates, and (2) the change of distance d between the plates. The first approach allows obtaining sensors with constant sensitivity (dC/dA= ɛ_0_∙ɛ_r_/*d*); the second one provides a non-linear relationship between C and *d*, with sensitivity decreasing with *d* (dC/d*d*= −ɛ_0_·ɛ_r_/*d*^2^)—where ɛ_0_ and ɛ_r_ are the permittivity of the free space and the relative permittivity of the dielectric, respectively. The characteristics of constant sensitivity offered by the first principium is attractive, on the other hand the second configuration requires easier design [40]. Although they require more sophisticated electronics than piezoresistive sensors, capacitive sensors are characterized by a good frequency response and a wide dynamic range. The microfabrication process allows them to be integrated in touch-sensitive surface with high spatial resolution. Their main drawbacks are the noise, in terms of crosstalk when arranged in the mesh configuration, the field interaction and the fringing capacitance: these disturbances need specific electronic to be filtered out [25].

## 3. Microfabrication Process

Microfabricated devices, also known as microelectromechanical systems (MEMS), are characterized by size ranging between micrometer and millimeter [40]. They can comprise movable parts, such as cantilevers, and fixed parts, such as flow channels and wells, chemically sensitive surfaces, for example biological components, like cells and biomolecules, and electrical part, like strain gauge.

Microfabrication is a fabrication process, composed by an ordered number of steps to build a physical object. Many methods and materials can be employed in this process, leading to the production of several products. The methods required for the manufacturing of the object are the following:

(1) photolithography: it is the process used for pattern transfer into the material. The pattern, designed by means of a CAD software, is transferred onto a glass mask, which has on the surface a photodefinable opaque material with the shape of the desired pattern. A substrate, spin-coated with photoresist (a photoresistive organic polymer), is placed in contact with the mask and they are hit by UV light, used to make the photoresist soluble into the opaque material. Lastly, mask and substrate are separated, and the photoresist is removed from the new system [41];
(2) stencil lithography: is a relatively new process used to produce patterns through a shadow mask and evaporation of material in a vacuum, and based on the method of physical vapor deposition. The main advantages of this method are the sub-micrometer resolution and its applicability with fragile substrates, like biological macromolecules [42,43];
(3) thin-film growth/deposition: thin films are largely employed in microdevices, could be made of various material, such as silicon, plastics, metals and, recently, biomulecules, and are formed by physical or chemical process, like sputter and chemical vapor deposition, or thermal oxidation [44,45];
(4) etching: it is the process of selectively removing materials in fixed patterns, using both liquid chemical substances (wet etching) and gas-phase chemistry (dry etching). Furthermore, etching can be either isotropic or anisotropic: in the first case, the etching acts equally in all direction of the space, whereas in the second case the effect is directional. Dry etching is commonly used to achieve anisotropic outcomes [45];
(5) bonding: the process of permanently binding together two substrates, in particular solid-state materials with smooth and flat substances, usually used for packaging. Many techniques have been developed to perform bonding, such as the fusion bonding, which employs chemical reaction between the bonding surfaces of several materials, and the anodic bonding, which is a thermally activated process supported by electrical field. Micromechanical sandwiched silicon systems are usually fabricated through high-temperature bonding (>700 °C), whereas silicon wafer and glass substrate are bonded together by means of middle temperature (200–500 °C) [46].

Two main processes are known: the bulk microfabrication and the surface microfabrication. The bulk microfabrication is characterized by etching and bonding of thick sheets of material such as silicon oxides and crystalline silicon. The surface microfabrication is based on the successive deposition and etching of thin films of material such as silicon nitride, silicon oxide and gold. Surface micromachining is one of the most common technologies used to manufacture MEMS sensors. In surface micromachining, films are deposited on a substrate and patterned, using photolithography, to create micromechanical devices. Most early surface micromachining used polycrystalline silicon (polysilicon) as the structural layers and an oxide of silicon as the sacrificial material. However, as surface micromachining has further developed, numerous other materials have been used. Depending on the desired application, MEMS developers have used metals, oxides and nitrides of silicon, and even polymers for both structural and sacrificial films [28].

The use of microfabricated sensors for medical applications introduces several advantages. The first consequence of miniaturization is the system integration: miniaturized devices can be easily housed within other tools, and allow embedding a number of units with different sensing principles within reduced space. In a microfabricated device the surface/volume ratio is generally high, allowing also low voltage supply and, as consequence, low power consumption. The reduced mass of components confers to MEMS-based sensors also attractive metrological properties, such as short response time and good dynamic response. The on-chip integration of electromechanical systems and the circuitry used to control them, allows further miniaturization. Lastly, microfabricated devices can be compound with polymeric and ceramic materials, which are attractive for biomedical applications due to their bio-compatibility, low cost, and suitability for rapid prototyping. [6,12,28].

## 4. Application in Medicine

### 4.1. Prosthetic Hands

The main requirements that a tactile sensor for prosthetic and robotic applications has to fulfill are the capability to estimate the magnitude and direction of the applied force, to distinguish the point of application of the force on the contact surface, to evaluate compliance and textural properties of manipulated objects, and to have a dynamic behavior comparable to the response of human mechanoreceptors in tracking tactile stimuli that vary with time. Especially in grasping and manipulation one fundamental requirement is to detect slippage in order to improve grasp stability and hand dexterity in manipulation tasks. In order to gain these features, general design criteria are based on the development of array sensors, with spatial resolution miming the one of human tactile system.

The spatial resolution required for tactile sensors depends on the location in human body. A number of receptors are embedded into the skin, associated to either myelinated or unmyeliated fibers: mechanoreceptors for pressure/vibration, thermalreceptors for temperature and nociceptors for pain/damage. Among these, mechanoreceptors mediate the response to mechanical stimuli, and are placed at different depths with respect to the skin epidermis, and with variable density on the human body. The most sensitive and highly populated areas are the hands' fingertips, the lips and the palms of both hands and feet. In particular, in an adult fingertips the average number of mechanoreceptors per square centimeter is about 240, whereas in the palm it is about 60 [47]. Mechanoreceptors are classified depending on their location with respect to the epidermis, affecting the extension of their receptive field, and the adaptation rate to static or dynamic stimuli. In the glabrous skin they are: Meissner’s corpuscles and Merkel’s cells (both type I receptors, surface-located), Ruffini corpuscles and Pacinian corpuscles (both type II receptors, deeply-located). Since the aim of this review is microfabricated tactile sensors in biomedical applications, some papers are advised for further information on the neurophysiology of human tactile system [48,49,50,51]. Nevertheless, when it is required to endow robots with a sense of touch, the features of the human tactile system should be taken as an example.

Although the human tactile system is complex, because it is not concentrated within one single organ and the functions are performed by several corpuscles and cells, the criteria of design of artificial tactile system take inspiration on the human one. The features that can be easily reproduced are the spatial resolution and the range of applied force. In particular, the criteria of formulation of tactile system in a generic robotic system are summarized by Dahiya [50] as following: the spatial resolution should be at least 1.6 mm in the case of fingertip [48] (an array of 10 × 15 elements on the surface) and less, *i.e.*, 5 mm in the case of palm; a force sensitivity range from 0.01 to 10 N is desirable, such as the feature to discriminate the direction of the force; the response time should be short, *i.e.*, 1 ms is a reasonable value to implement real time conditions; sensors in array configuration can be covered with an elastic and skin-like membrane, which can be designed in order to concentrate the stress on the sensing element; metrological characteristics, such as monotonicity and low hysteresis are also desired.

It is worth observing that tactile sensors are intended such sensors that acquire information through the physical contact. Hence, other physical quantities and properties, such as temperature [52,53], slip [54,55], vibration [56], shape and texture [57,58] can also be measured by tactile sensors.

One of the first designers of microfabricated sensors was Hillis in 1981 [1]. His array of 256 tactile sensors was intended to be used on a robotic finger: each sensor was characterized by a surface of about 0.01 cm^2^, and had a measurement range between 0.01 and 1 N. The principle of sensing of this sensor lies outside of the principles described in Section 2, but it is here proposed as an example of the first microfabricated sensor. The sensor array was composed of a flexible printed circuit board and a sheet of conductive silicon rubber covered with high conductive material (silver or graphite), with a separator of non-conductive material (nylon). The applied force on the silicon rubber deforms the material around the separator, allowing the contact between the silicon and the metal below, and increasing the conductance of the device. Since the resistance is inversely proportional to the contact area, the higher the applied force, the higher the deformation and, therefore, the contact area between silicon and metal. The relationship between applied force and conductance of contact is not linear, but shows a higher sensitivity in correspondence of lower forces (between 0.05 and 0.25 N the sensitivity is about 0.025 mN·Ω, whereas it halves to 0.012 mN·Ω for bigger values of force). This prototype was used to assess the capability of the robot finger to recognize the shape and orientation of different objects.

#### 4.1.1. Piezoresistive Sensors

In 1995, Beebe *et al*. presented their work on a silicon-based microfabricated force sensor for medical and robotic purposes [59]. The sensitive element was based on a silicon piezoresistive diaphragm sensor, where the silicon is bonded onto a glass substrate to form the reference chamber. The applied force is transmitted to the diaphragm by a solid dome, which transforms the load into pressure on its surface. Under load the diaphragm deforms, inducing a change in resistance of the four piezoresitors used in Wheatstone bridge configuration. The sensitive element, packaged in skin-like polyimide, has a thickness of 710 µm and an edge of about 10 mm. Two different materials, epoxy and Torlon, have been used to fabricate the domes. Epoxy dome showed a significant hysteresis, therefore Torlon has been preferred. The sensor output is linear for force values lower than 10 N. In 1998, the same sensor was mounted onto the thumb of five volunteers, to investigate the sensor's performances in a realistic scenario [60].

**Figure 1:**
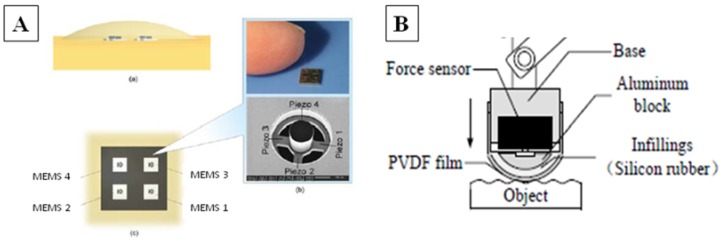
(**A**) Piezoresistive array sensor for robotic finger [63] and (**B**) finger-shaped piezoelectric tactile sensor [37].

A huge effort in the development of microfabricated tactile sensors array to be implanted within prosthetic hand has been made by the research group headed by Dario [61,62]. The sensor consists of a flexible sensing structure with four tethers whose axes are perpendicular to each other in a cross-shape, and a cylindrical mesa, located at the cross center, that transmits the force (Figure 1A). Four piezoresistors convert the stress into change of resistance, and Wheatstone bridge configuration is used to obtain the voltage output, which is related to the change of resistance. Each sensor has the dimensions 2.3 mm × 2.3 mm × 1.3 mm, is arranged in an array of 4 × 4 (with a total of 16 sensors) and encapsulated within polyurethane material. The average sensitivity of each piezoresistor is 0.032 N^−1^ for an applied normal force between 0 and 2.4 N, whereas is values 0.054 N^−1^ for the tangential load between 0 and 0.4 N, with a linearity of 99.7%. The breaking normal load is around 3 N and the breaking shear load ranges from 0.5 to 0.7 N. The 4 × 4 array has been encapsulated in an artificial skin-like material—*i.e*., PDMS—and mounted on the distal phalanx of a robotic finger. The biomimetic system underwent tests for roughness encoding by means of a sliding platform simulating texture related vibrations with spatial periodicity from 400 to 1900 μm, constant speeds of sliding (from 5 to 40 mm/s) under regulated normal contact forces (between 100 and 400 mN). In further works, the same group presents the characterization of the array to be employed for the passive [62] and active-touch [63] classification of textures, emulating the behavior of an active underactuated robotic finger as well as machine learning strategies [64] in exploring objects.

#### 4.1.2. Piezoelectric Sensors

One of the first tactile sensor based on piezoelectric effect was developed by Ando and Shinoda [65] in 1994. The principle of measurement of this tactile transducer is based on acoustic ultrasonic sensing. In particular, a 2 × 2 matrix of electrodes on PVDF layer is housed within a silicon finger-like body: when the silicon surface is touched, waves are transmitted to the PVDF, which detects the ultrasonic emission caused by touch and slip. The authors claimed a good spatial resolution (around 2.5 mm) and high temporal resolution.

Since 1995, Dargahi started a deep investigation of PVDF film with piezoelectric behavior to be employed in thin touch-sensing sheets. Although his main aims were in the field of micro-surgery tools (described in Section 4.2), the author proposed a 25 µm thick PVDF membrane with three sensitive electrodes, used to discriminate the position of force applied by probes, with a resolution less than 5 mm [66].

In 2005 Choi *et al*., proposed a miniaturized PVDF sensor to be housed on finger and thumb tips of the SKKU Hand II [67]. A matrix of 24 sensing elements, with size 0.5 mm × 0.5 mm, constitutes the flexible sensitive layer [68]. Since PVDF is adequate for sensing dynamic force, it has been used to measure slip; in order to perform also measurement of force, the PVDF matrix has been coupled with a pressure variable resistor ink, made by electrically conductive ink whose resistance decreases with increasing applied force. The combined sensor has been proved to detect clearly the rolling of an object with mass 100 g, such as the static load of masses of 100 g and 200 g, with a constant sensitivity of about 20 mV·g^−1^.

#### 4.1.3. Capacitive Sensors

One of the first microfabricated tactile sensors based on capacitive principle of working has been presented by Gray and Fearing in 1996 [69]. Although the authors did not describe a specific application of their sensor, the project criteria are based on specific requirements suitable for biomedical purpose. A rubber layer distributes the forces on the surface of a polysilicon capacitive array, housing sensing elements with width size of about 90 µm. The mean sensitivity, Sc, of the sensor, expressed as

(3) $$S_{C}=\frac{\%\Delta C/C}{\Delta F}$$

was found to be 0.005% µN^−1^, with a discrimination threshold of 20 µN and 2.3 kPa. The authors reported that hysteresis is the main concern of the sensor, attributed to manufacturing defects.

**Figure 2:**
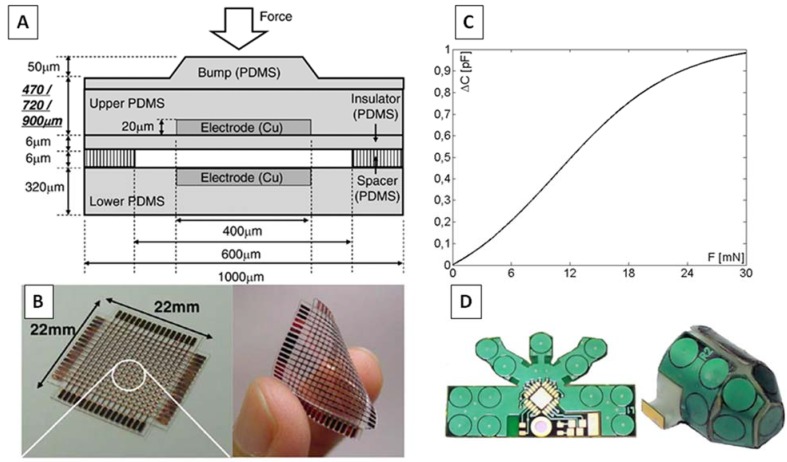
Capacitive sensors used for prosthetic and robotic hands: (**A**) and (**B**) Capacitive tactile sensor consisting of 16 × 16 tactile cells and using polydimethylsiloxsane (PDMS) elastomer [71]; (**C**) typical relationship between change of capacitance and applied force in bio-inspired MEMS sensor; (**D**) Flexible pressure sensor system designed for the fingertips of the humanoid robot iCub [72].

In 2005, Lee *et al*., proposed a modular expandable capacitive tactile sensor consisting of 16 × 16 tactile cells, using polydimethylsiloxsane (PDMS) elastomer [70,71]. The module is characterized by 1 mm spatial resolution: each cell has dimensions of 600 µm × 600 µm and initial capacitance of 180 fF. The cell is composed of five PDMS layers, and copper electrodes are embedded in the PDMS membrane (Figure 2A,B). The sensing capacitor is arranged between the two electrodes. When pressure is applied to a bump, the upper PDMS deforms and the capacitance increases until the air gap is completely closed. The sensor sensitivity, which depends on the total thickness of the upper electrode and bump layer, is 3% mN^−1^ within the whole measurement range of 40 mN (250 kPa). The expandability of the flexible sensor has been demonstrated, by realizing a 32 × 32 array by using a conductive paste to connect the four above-described modules.

An artificial skin for robots has been developed, based on capacitive sensors with a triangular shape. Although the low power consumption (5 W·m^−1^) and the feasibility to conform with complex surfaces, the sensor matrix is characterized by a low spatial resolution (about 2 sensitive elements per centimeter) [73]. Based on this principle, a flexible pressure sensor system has been designed for the fingertips of the humanoid robot iCub. A flexible printed circuit board (PCB) embeds 12 circular patches, acting as the fixed conductors of capacitors. The external force acts on an external deformable conductor layer, which is separated from the PCB by a dielectric silicon rubber foam (Figure 2C). The main drawback of this sensor is the high hysteresis (about 25%), ascribed to the silicon foam [72].

In 2011 Muhammad *et al*., proposed a bioinspired MEMS capacitive sensor array [74], whose individual sensing element consists of an upper 2 µm highly doped single crystal silicon diaphragm, a 2 µm air cavity and a lower electrode consisting of highly doped silicon. Each element has a size of 500 µm × 400 µm, and the distance between the elements is 150 µm. Reference capacitors, not subjected to pressure, have been embedded in the array, in order to achieve differential response and eliminate effects of parasitic capacitance. The sensor system has been covered with PDMS. The relationship between the applied force and the change of capacitance is shown in Figure 2D. The average sensitivity of the bare sensor is 0.035 pF/mN within the measurement range up to 25 mN; the use of PDMS entails a decrease of sensitivity (0.068 fF/mN), but allows a wider measurement range (up to 1.7 N). The same system has been tested to discriminate texture by scanning different surfaces, consisting on nylon and polycotton fabrics, as well as on irregular texture patterns, with gratings varying in spatial periodicity from 400 to 1200 µm and tangentially scanned with velocities ranging from 0.05 mm·s^−1^ to 4 mm·s^−1^ [75].

### 4.2. Microsurgical Force Sensors

A significant research effort has been made since the 1990s to develop tools for minimal access surgery. It includes minimally invasive surgery (MIS), endoscopic and laparoscopic surgery, as well as robotic-assisted minimally invasive surgery. The advantages offered by these surgical approaches over conventional operations include reductions in intraoperative blood loss, tissue trauma, risk of post-operative infection, pain experienced by the patient and recovery time [76,77]. However, the reduced invasiveness of the procedures are conducted at the expense of some issues for the surgeons, such as the constrained spaces due to key-hole incisions and the reduction in the degree-of-freedom during manipulation, and the lack of haptic feedback during the tool-tissue interaction [78]. In order to provide information about different properties of tissues undergoing minimal access surgery, surgical tools are equipped with tactile sensing systems, which includes by three main parts: (i) a tactile sensor, which transduces the contact force in an electrical quantity; (ii) a platform of signal processing and (iii) a part that displays the processed data to the clinician. In this review, we focus on the first part, with particular attention to the most commonly used microfabricated tactile sensors.

During tool-tissue interaction, tactile information should include: the amplitude of contact force, the distributed force information, the degree of hardness for the contact tissue, and the local discontinuities in the hardness of contact tissue.

In general, force tactile sensors employed in surgical applications are based on mechanical indentations on the tissue: they record force response with respect to indentation depth in order to get information about the stiffness of the soft tissues by measuring feedback force from the target material. Specific review papers are recommended to obtain more details about the complete system and the integration in surgical tools [76,78,79].

#### 4.2.1. Piezoelectric Sensors

Several microfabricated tactile sensors for minimal access surgery are based on piezoelectric technology. The first example of a microfabricated tactile sensor employed for MIS was described by Eklund [80], who used a PZT crystal at the tip of a catheter to estimate the hardness of *in vitro* human tissue. The sensor’s principle of measurement was based on the dependence of oscillation frequency of the crystal on the hardness of the target.

Dargahi and colleagues investigated the benefit of piezoelectric tactile sensors for MIS purposes [66,81,82]. They proposed a tooth-like patterned silicon layer, which transmits the forces to an underlying PVDF layer, stuck on a substrate of Poly(methyl methacrylate). The system has a spatial resolution of 3 mm. Modification of the first prototype allowed enhancing spatial resolution [83].

Ezhilvalavan *et al*. proposed a piezoelectric sensor, intended to be housed in MIS tools. The authors focused especially on the fabrication process, based on the use of lead zirconate titanate (PZT). In particular, the PZT sensor is a parallel plate capacitor structure in which the 1-μm thick PZT film is sandwiched between top (Au/Cr) and bottom (Pt/Ti) metal electrodes mounted on a thin Si membrane. The main feature of this sensor is to be "free-standing", because the sensor is totally free from substrate. This feature differs the above described sensor from the other microfabricated PZT sensors, which are usually supported on a Si substrate in the form of cantilever structures. The sensor proposed by Ezhilvalavan aimed to overcomes the issue of substrate clamping effect in distorting the modes of displacements [84].

**Figure 3:**
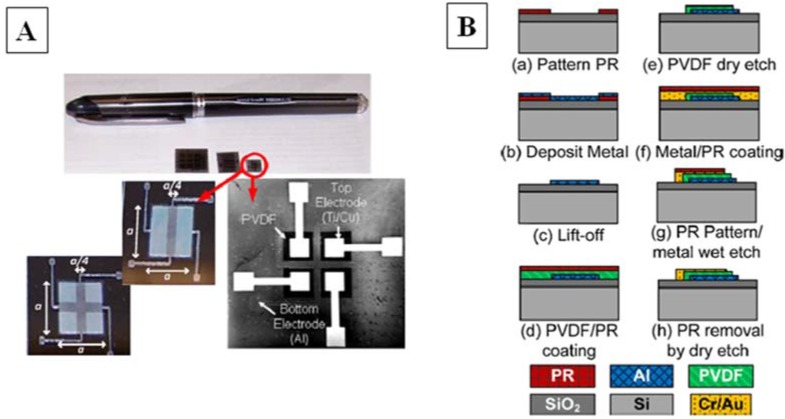
(**A**) Pictures and (**B**) fabrication process of film PVDF–TrFE based pressure sensor for catheter application [85].

Attention has been directed to the optimization of process and steps of microfabrication, such as to more sensitive materials. Among these, polyvynildene fluoride trifluorethylene (PVDF-Tr), which is a co-polymer of PVDF, has been demonstrated to have excellent piezoelectric properties [85,86] (Figure 3). Li *et al*. [86] fabricated flexible tactile sensors to be housed in smart microcatheter, with a minimum thickness of 500 µm. The voltage output is directly proportional to the external force applied to the thin film surface, with a sensitivity of about 1 V·mN^−1^. The absence of the temperature effect on the sensitivity of the sensors has also been assessed and the discrimination threshold is 25 mN.

#### 4.2.2. Piezoresistive and Capacitive Sensors

The use of piezoresistive microfabricated devices for tactile sensing in minimal access surgery has been investigated by few research groups [87,88,89], and poor is the literature regarding capacitive sensors for this purpose. In 2006, Valdastri *et al*., proposed the miniaturized tri-axial force sensor described in [61] to be used as force sensor system in minimally invasive surgical tool [90]. In 2010, Ahmadi *et al*. [89] proposed a hybrid microfabricated catheter-tip sensor, based on both optical fiber and piezoresistive effects, used to measure relative hardness of contact tissues during surgical mitral valve repair.

### 4.3. Biomechanical Analysis

Since the 1990s, a significant research effort has been made to develop sensors for biomedical applications such as in-shoe elements, to measure the foot pressure during gait analysis [91], or to monitor contacting forces between stump and prosthesis. All of these applications can be referred to as the macro-area of biomechanical analysis. 

#### Piezoresistive Sensors

In 2000, Hseih *et al*. [92] proposed a microfabricated shear stress sensor, to measure the contact stress between skin of stump and socket of above-knee prosthesis. The aim of this study was to investigate the skin surface friction, which may damage the tissue and affect their normal function. A silicon membrane of 3000 × 3000 × 300 µm^3^ was provided with a small flange on its top, and a couple of two piezoresistors, with angle of 90° between them and perpendicular to the flange surface. When subjected to shear force, the flange causes a normal load on one piezoresistor and, at the same time, a shear stress on the other one. The sensor has a linear response with sensitivity of 0.13 mV·mA^−1^·MPa^−1^ in the range 0–1.4 N, and shows a mean hysteresis error of 3.5%.

In 2009, Alfaro *et al.* [93] proposed an implantable MEMS sensor aimed to monitor the intraosseous bone stress based on piezoresistive technology, similar to the one described in [92]. The wireless microminiature intraosseous sensor system for measuring multi-axis stresses at the microscale includes a central MEMS transducer array, a surrounding coil antenna for wireless operation, and electronics, all integrated on a single 3 mm × 3 mm CMOS chip. The transducer array has a 1 mm × 1 mm footprint and is an 8 × 8 array of piezoresistive strain gauges. Texturing of the silicon CMOS chip surface into 60 μm × 60 μm × 60 μm tall posts will help to enhance cell growth and osteointegration. For loads ranging from 30 to 400 kPa, the mean sensitivity before amplification was found to be 190 μV·V^−1^·MPa^−1^, and the mean hysteresis error around 10% of full scale.

A prominent contribution to the development of microfabricated tactile arrays for smart interfaces for biomechanical measurements has been made by the group founded by Dario and Carrozza [61,94]. The sensor consists of a flexible sensing structure with four tethers whose axes are perpendicular to each other in a cross-shape, and a cylindrical mesa, located at the cross center, that transmits the force. Four piezoresistors convert the stress into change of resistance, and Wheatstone bridge configuration is used to obtain the correspondent voltage output. Each sensor has dimensions of 2.3 mm × 2.3 mm × 1.3 mm, is arranged in an array of 4 × 4 and encapsulated within polyurethane material. For an applied normal force between 0 and 2.4 N, the average sensitivity of each piezoresistor is 0.026 N^−1^, whereas 0.054 N^−1^ for tangential load between 0 and 0.4 N, with a linearity of 99.7%. The breaking normal load is around 3 N and the breaking shear load ranges from 0.5 to 0.7 N.

In 2008, Wahab *et al*., proposed a micro-sensor for measurement of foot pressure during gait. Four piezo-resistors are placed under a deformable membrane, which is in contact with the foot sole. The voltage output of the sensor system, provided by a Wheatstone bridge configuration of the piezo-resistors, is proportional to the applied pressure, in the range 0–2 MPa [95].

### 4.4. Multimodal Sensors

A tactile sensor provides information about properties of the object trough the physical contact. Among these, temperature, texture, slips, as well as force and pressure, are useful information to obtain during contact. The majority of the studies in the literature so far reviewed and discussed, focused on the development of microfabricated sensors or systems sensitive to one specific physical quantity (mostly force and pressure sensors).

**Figure 4:**
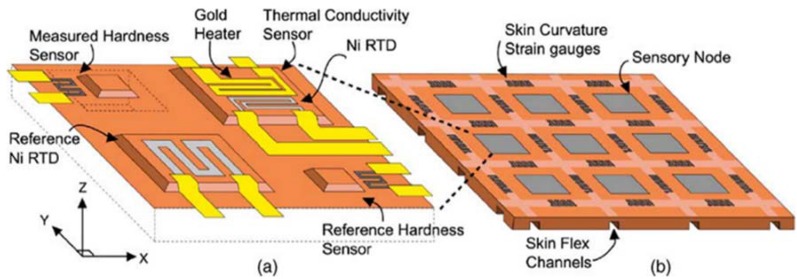
Multimodal sensor performing measurements of hardness, temperature, thermal conductivity and curvature of object [97].

Along with the growing interest on tactile sensing in many field of medicine and industrial applications, the need for developing a system that can provide information about two or more properties is increasing. These systems are known as multimodal sensors, and some relevant examples are reported in the following. In 2002, Castelli integrated capacitive tactile sensors with resistive thermal sensor, obtaining an array of 8 × 8 with pressure sensitivity of 0.05 pF·MPa^−1^ in the range 0–120 MPa and spatial resolution of about 2 mm, as well as thermal sensitivity of 40 mΩ·°C^−1^ [52]. Although using different materials, *i.e*., polymide and copper layer for tactile sensor, and commercial analog temperature sensor, Yang *et al*., proposed a highly flexible multimodal sensor 10 × 10 array, to be used as artificial skin for robots [96]. In 2005, Egel *et al*., showed a multimodal microfabricated sensor system, used to measure temperature, thermal conductivity, hardness and surface curvature of the explored object, thanks to the integration of different sensors (Figure 4): a resistance thermometer RTD for temperature and thermal conductivity, a sensor based on membrane deflection for the hardness, strain gauges for the curvature [97].

### 4.5. New Frontiers

Although the aim of this paper is to provide an overview of microfabricated tactile sensors, it is worth mentioning the other sensing principles, which allow designing micro-sensors for tactile purposes.

The traditional working principles of tactile sensors continue to be widely used and investigated; however, new frontiers lead to investigate novel phenomena, or to borrow alternative techniques from other fields of engineering. An example is provided by optics, fluidics, resonance sensors, and ionic polymer metal composites.

Concerning optics, several principles are employed to develop sensors, mostly used in MIS, in particular sensors based on the light intensity modulation technique, interferometry and Bragg grating technology. Fiber-optic based sensors are very attractive for many applications of medical field, such as the immunity to electromagnetic interference and MRI compatibility, biocompatibility, non-toxicity and chemical inertness, robustness, flexibility, high versatility and the feasibility of being miniaturized [97,98,99].

**Figure 5:**
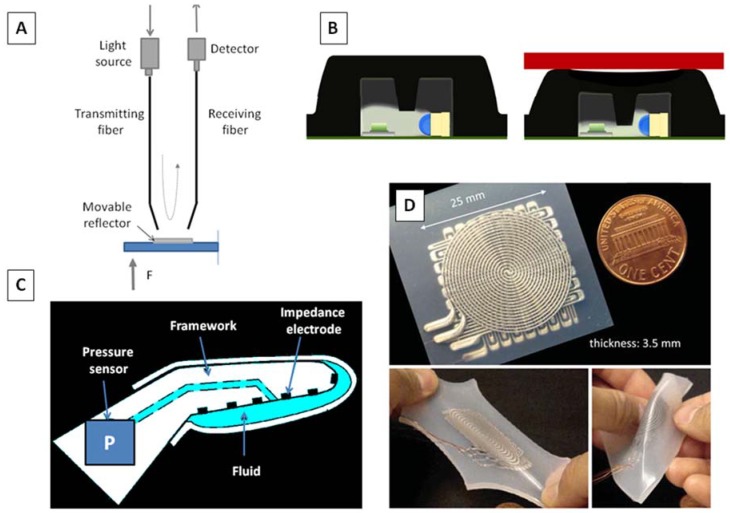
(**A**) Example of fiber-optic based pressure sensor for minimally invasive surgery; (**B**) in-shoe optomechanical transducer of foot pressure [109]; (**C**) fluidic (readapted from [55]) and (**D**) microfluidic tactile sensors for artificial skin [112].

Fiber optic tactile sensors are mainly used with tools for MIS and endoscopy, with different principles of sensing: the general approach considers the use of at least a couple of fibers, the former transmitting light on a mobile and reflecting structure, and the latter receiving the light intensity signal reflected by the structure. When the force dislocates the mechanical structure, the intensity of light passing through the receiving fiber changes, as shown in Figure 5A [100,101,102].

In addition, techniques based on the Fabry-Perot interferometer (FPI) have shown great promise in this area. For instance, a miniaturized fiber optic tactile force sensors based on FPI has been proposed to provide tactile feedback and measure the forces of interactions during needle-based percutaneous procedures in an MRI, or in vitreoretinal microsurgery applications [103,104].

Since 2006, fiber Bragg gratings (FBGs)—optical elements with minimum size in the order of 1 mm—are also deeply investigated to be housed in microsurgical tools, catheters and needle biopsy [102]. The advantages of FBG are the use of the absolute wavelength as a sensing signal which avoids the problems of light intensity fluctuations, which affects the above-described optical technologies, and the ease of housing several gratings within the same fiber. The main drawback is related to the simultaneous sensitivity to strain and temperature, which can be compensated by using reference temperature sensors. Some applications of FBG for the design of distributed tactile sensors for miming skin-like surfaces have been shown [105,106].

Other principles of sensing are based on the light modulation induced by relative displacement of two micro-fabricated gratings [107], and on a couple pair photoemitter-photoreceiver, where the intensity of received light depends on the displacement of an intermediate shadowing element (Figure 5B) [108].

Concerning the employment of fluids in tactile sensors, the main application field is prosthetics. A deformable fluid housed within an elastomeric skin can be used to sense micro-vibration during contact events. It has the advantage of being highly sensitive to normal and shear forces [56,109].

For instance, in the work of Fishel *et al*. [56], a PDMS skin having microchannels filled with a liquid metal alloy was wrapped around a human finger. Deformation induced changes in resistance of the fluidic electrical circuit to measure joint angles when the finger was bent (Figure 5C).

In a recent study, Ponce Wong *et al.* proposed a flexible, capacitive, microfluidic sensor for normal force sensing with microchannels. The microchannels act for both flexible wire paths and conductive metal plates of the capacitive sensing units [110]. The 5 × 5 array has sensor elements with dimensions of 0.5 mm × 0.5 mm. The main advantage of fluidic sensors over the standard solid materials are related to the robustness, as well as to the flexibility and deformability that allow them to be easily embedded in artificial skin (Figure 5D) [111]. Other emerging trends are neuromorphic coding of tactile information [112,113] and integration of living cells in the mechanotransduction chain [114].

Resonance sensors are also employed in the tactile sensing field. They are based on the frequency shift between the resonance frequency of a freely vibrating sensor and the one measured when the sensor makes contact to an object. The frequency shift depends on the acoustic impedance of the object and can be used to characterize its properties [115,116].

Among many biomedical applications, resonance sensors have been proposed for the measurement of tissue elasticity in the field of human assisted reproductive technology, to determine the change of stiffness of the human ovum during the sequence of fertilization [117], or for prostate cancer detection [118].

Lastly, electroactive polymers are gaining interest for tactile sensing applications. When a stimulus is applied to these polymers, they change their voltage output or their shape. PVDF, already discussed in Section 2.2, and ionic polymer metal composites (IPMCs) belong to the family of electroactive polymers [119]. In particular, IPMCs are based on the shift of mobile charges induced by deformation, and are known for developing actuators and artificial muscles, since they show significant deformation in presence of low applied voltage (actuation displacement of more than 10%) [120]. IPMC are under investigation for the design of tactile sensors to measure pressure distribution within human spine [121], or for minimally invasive surgery purposes [122]. In addition to the valuable features of being easily miniaturized and high sensitivity to strain, they can be used in wet environment, therefore compatible with endoscopic and biological scenarios.

## 5. Discussion and Conclusions

This review presents an analysis of microfabricated technologies for developing tactile sensors destined to the medical field.

Table 1 summarizes all the technologies investigated within this overview, focusing on the microfabrication process, design, application and, when showed by the authors, the metrological properties of the sensor.

Tactile sensing is required when tools and devices are used to perform tasks carried out by humans that in daily activities. Human touch needs to be replaced in some specific contexts, e.g., when high dexterity and accuracy are required in procedures operating in a non-human scale (*i.e*., minimal access surgery), or else, when tactile interfaces are inaccessible and biomechanical properties have to be monitored (*i.e*., smart interface), or else when haptic feedback and the perception of the interaction with the external environment is required (*i.e*., prosthetics and artificial skin).

**Table 1:** Sensing principle, microfabrication process, design, applications and metrological properties of tactile sensors.

Sensing Principle	Author, Year, Reference	Microfabrication Process	Design	Application	Metrological Properties
Piezoresistive sensors	Beebe *et al*., 1995–1998 [59,60]	Silicon direct bonding and bulk micromachining	Silicon piezoresistive diaphragm	Human finger force measurement	- Linearity up to 10 N - sensitivity in linear range 16 mV∙N^−1^
	Dario, Carrozza *et al*., 2005–2009 [61,62,63,64,90,94]	Subtractive dry etching	Silicon-based three-axial force sensor	Robotic tactile sensing; MIS	- 2 × 2 array - measurement range up to 2 N - sensitivity 0.032 ± 0.001 N^−1^ - artificial roughness encoding
	Dargahi *et al*., 2010–2011 [88,89]	-	PVDF membrane	MIS	- Measurement range up to 25 N - sensitivity 10 N∙m^−1^ - resolution 0.1 N - repeatability 2.5% full scale
	Hseih *et al.,* 2000, [92]	Silicon bulk micromachining	Micro shear-stress sensor	Biomechanical analysis	- Measurement range up to 1.4 N - sensitivity 0.13 mV·mA^−1^·MPa^−1^ - mean hysteresis error of 3.5% of full scale
	Alfaro *et al*., 2009, [93]	CMOS process, maskless dryetching	Piezoresistive strain gauges	Biomechanical analysis	- 8 × 8 array - measurement range 30–400 kPa - mean sensitivity 190 μV·V^−1^·MPa^−1^ - mean hysteresis error of 10% of full scale
	Wahab *et al*., 2008, [95]	Silicon bulk processing (designed only)	Wheatstone bridge configuration	Biomechanical analysis	- Theoretical measurement range up to 2 MPa - theoretical sensitivity 20 mV∙MPa^−1^
	Ando *et al*., 1994 [65]	Etching	PVDF electrodes housed in silica	Artificial tactile sensing for touch and slip	- 2 × 2 array - resolution 2.5 mm
	Dargahi *et al*. [66,81,82,83]	Photolithography and anisotropic etching	Silicon, tooth-like pattered layer transfers force to PVDF film	Endoscopic grasper	- Measurement range up to 2 N - sensitivity 100 mV·N^−1^
	Ezhilvalavan *et al*., 2006 [84]	Deep reactive ion, ion beam and wet-chemical etching	PZT force sensors with top and bottom electrodes forming capacitor	MIS	Only electrical characterization, e.g., leakage current 10^−7^ A/cm^2^ (applied electric field of 200 kV·cm^−1^)
	Li *et al*., 2008 [86]Sharma *et al*.,2012 [85]	Mold-transfer method	PVDF-TrFE copolymer	MIS	- Measurement range up to 1 N - sensitivity 10 mV∙N^−1^ - bandwidth 0–100 Hz - discrimination threshold 25 mN
Capacitive sensors	Gray and Fearing 1996 [69]	-	Rubber layer on polysilicon capacitor	General biomedical purposes	- Sensitivity 0.005% μN^−1^ - discrimination threshold 20 μN
	Lee *et al*., 2005–2006 [70,71]	Bonding	PDMS layer	Robotic skin	- 16 × 16 array - measurement range up to 40 mN (250 kPa) - sensitivity 3% mN^−1^ - spatial resolution 1 mm
	Muhammad *et al*., 2011 [73,75]	Bonded and Etched-Back Silicon-On-Insulator wafers, Deep Reactive Ion Etching	PDMS-coated capacitive sensor	Robotic finger	- 1 × 4 array - measurement range up to 1.7 N - sensitivity 0.068 fF·mN^−1^ - artificial roughness encoding
Multimodal sensors	Castelli 2002 [52]	-	Capacitive sensors for force and temperature	Robotic tactile skin	- 8 × 8 array - pressure range up to 0.25 N/mm - pressure sensitivity 0.05 pF·N^−1^·mm^2^ - force range up to 81 N - temperature range up to 150 °C - thermal sensitivity 0.4 mΩ·°C
	Egel *et al*., 2005 [97]	Etching, lift-off pattering	Strain gauge for force measurement, RTD for temperature measurement	Robotic tactile skin	-
Optical-based sensors	Su *et al*., 2011 [104] Liu *et al*., 2012 [105]	-	Fabry-Perot interferometer	MIS	- Measurement range up to 10 N - Mean sensitivity −40 mV·mɛ^−1^
	Cowie *et al*., 2007 [107]	-	Fiber Bragg gratings	General biomedical purposes	- 3 × 3 array
	De Rossi *et al*., 2001 [109]	-	Light intensity modulation	Biomechanical analysis	- Measurement range up to 50 N - Mean sensitivity −0.02 V·N^−1^
	Ahmadi *et al*., 2010 [89]	-	Light intensity modulation	MIS	-
Fluidic sensors	Fishel *et al*., 2008 [56]	-	Pressure sensor housed into a fluid-filled fingertip	Biomimetic Fingertips	-
	Ponce Wong *et al*., 2012 [111]	Soft lithography	Galinstan-filled microchannels	Artificial skin	- 5 × 5 array - measurement range up to 2.5 N - spatial resolution 0.5 mm
	Park *et al*., 2012 [112]	Silicon layered molding and casting process	Multilayered mircochannels in elastomer matrix	Fingertips	-
Ionic Polymeric Metal Composite (IPMC)	Bonomo *et al*., 2008 [122]	-	Two IPMC membranes	MIS	- Measurement range 100–300 Pa - mean sensitivity 200 m∙VPa^−1^

Among the several technologies and principles of sensing, piezoresistor materials are the most widespread in the fields of both prosthetics and smart interfaces. This is due to the nature of piezoresistive material, which allows microfabrication of flexible and compliant layers. On the other hand, piezoelecticity is at the base of working principle of a number of microfabricated tactile sensors employed for artificial skin and microsurgical and endoscopic tools, whereas capacitive sensors are widespread in prosthetic applications. Different technologies can also be integrated in a unique system, aimed to perform multimodal measurement of many contact parameters with a reduced occupied encumbrance and increased portability.

From the analysis of literature performed by this review, it emerges that microfabrication is the *sine qua non* condition for the design and development of performing sensors for tactile purposes in many biomedical applications. Nevertheless, new principles for working and designs are emerging, involving fiber optics, microfluidic devices and materials, like IMCP. All of them are gathering a huge interest from a number of research groups because of relevant features, such as immunity from electrical field interferences, the compatibility with MRI and wet environment, which overcome the classical characteristics of MEMS. Therefore, the growing and continuous research in the field of tactile sensing for biomedical application will go towards the fusion of many technologies, aiming to enhance the pros of each technique.
